# Development of Triptolide Self-Microemulsifying Drug Delivery System and Its Anti-tumor Effect on Gastric Cancer Xenografts

**DOI:** 10.3389/fonc.2019.00978

**Published:** 2019-10-03

**Authors:** Minghua Xie, Jia Wu, Liqaing Ji, Xiaorui Jiang, Jin Zhang, Min Ge, Xinjun Cai

**Affiliations:** ^1^Department of Pharmacy, First People's Hospital of Yuhang District, Hangzhou, China; ^2^Department of Pharmacy, Zhejiang Integrated Traditional Chinese and Western Medicine Hospital, Hangzhou, China

**Keywords:** triptolide, self-microemulsifying delivery system, central composite design and response surface methodology, MGC80-3 cells, anti-tumor effect

## Abstract

**Purpose:** To develop a triptolide (TP) self-microemulsifying drug delivery system and to investigate its anti-tumor effect on human gastric cancer line MGC80-3 xenografts in nude mice.

**Methods:** The medium chain triglyceride (MCT) was selected as oil phase; polyoxyethylene castor oil (EL) was selected as surfactant, and PEG-400 was selected as cosurfactant. The mass ratio of each phase was optimized by central composite design and response surface methodology to prepare TP-SMEDDS (self-microemulsifying drug delivery system). The quality of TP-SMEDDS was evaluated, and its inhibitory effect on tumor growth investigated in nude mice transplanted with MGC80-3 cells.

**Results:** The final prescription process was defined as follows: MCT mass ratio: 25.3%; EL mass ratio: 49.6%; PEG-400 mass ratio: 25.1%. The prepared TP-SMEDDS was a transparent liquid with a clear appearance (the theoretical particle size: 31.168 nm). On transmission electron microscopy, the microemulsion particles were spherical in size and uniformly distributed without adhesions. The *in vitro* release experiment showed complete release of the prepared TP-SMEDDS in PBS solution in 6 h. *In vivo* antitumor activity showed its inhibitory effect in the xenograft model.

**Conclusion:** The self-microemulsifying delivery system improved the oral bioavailability and the *in vivo* antitumor effect of TP.

## Introduction

**Figure 1:**
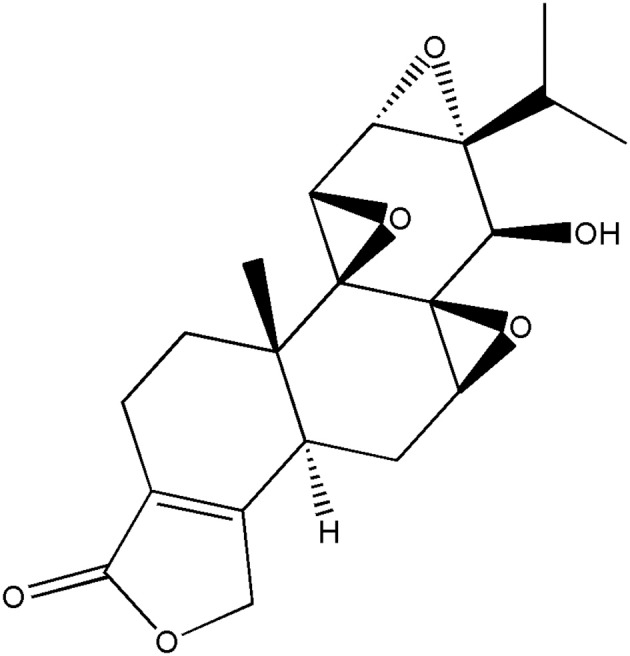
The structure of triptolide.

Triptolide (TP) is a type of epoxide diterpene lactone isolated from *Tripterygium wilfordii* and other plants its structure is as shown in Figure 1. It has a wide spectrum of anti-tumor effects and exhibits strong inhibitory effect on more than 60 tumor cell lines (1–3). Its anti-tumor effect is mediated via multiple mechanisms and targets, and is stronger than that of traditional anti-tumor drugs such as cisplatin and paclitaxel. Tumor cells that are resistant to traditional antitumor drugs have been shown to be very sensitive to TP (4–9). Triptolide is a potential antitumor drug. However, it has low water solubility, poor bioavailability *in vivo*, and causes severe gastrointestinal irritation. The toxic and side effects of TP limit its clinical application.

Self-microemulsifying drug delivery systems (SMEDDS) refer to a solid or liquid formulation containing oil phases, surfactants, and cosurfactants (10, 11). The O/W microemulsion (particle size ≤ 100 nm) can spontaneously form in the gastrointestinal tract or on slight stirring at 37°C (12–15). SMEDDS represents a new oral drug delivery system that can increase the solubility of water-insoluble drugs and improve the oral absorption and bioavailability of drugs (16, 17). It also reduces the cytotoxicity of drugs administered as high-concentration aqueous solutions and avoids the hydrolysis of water-unstable drugs and gastrointestinal irritation caused by drugs (18). Besides, self-microemulsion offers great advantages in improving drug solubility and bioavailability and in reducing gastrointestinal adverse effects (19–21).

SMEDDS is characterized by a simple preparation process and convenient oral administration of auxiliary materials; moreover, it has non-irritating odor. Intergration of TP into SMEDDS will help retain its antitumor activity, improve its water solubility, reduce its toxicity, and provide a theoretical basis for expanding the clinical application of triptolide. In this study, we optimized the prescription process of TP self-microemulsion using central composite design and response surface methodology. In addition, we evaluated the appearance, average particle size, morphology, and distribution of self-microemulsion. Lastly, we investigated the *in vivo* anti-tumor effect of TP in a MGC80-3 xenograft model.

## Materials and Methods

### Materials

Triptolide (TP) was purchased from Shanxi Pioneer Biotech Co. Ltd. (Shanxi, China, purity > 99%). Middle-chain triglyceride (MCT) was purchased from Shanxi Pioneer Biotech Co. Ltd. (Xian, China, purity > 98%). Polyethylene-polyoxyethylene-oil was purchased from Shanxi Pioneer Biotech Co. Ltd. (Xian, China, purity > 98%). PEG was purchased from Shanxi Pioneer Biotech Co. Ltd. (Xian, China, purity > 98%).

Cancer cell line MGC80-3 was purchased from China Center for Type Culture Collection (Wuhan, China).

BALB/c nude mice (6–8 weeks) weighing 18–20 g were obtained from the Shanghai Slac Laboratory Animal Co. Ltd. Animal certificate: SCXK (Shanghai) 2007–0005. The animal experiments in this study were performed under the guidance of the care and use of laboratory animals in Zhejiang University (Hangzhou, China) and conformed to the National Institutes of Health Guide for Care and Use of Laboratory Animals (Publication No. 85–23). The temperature in the SPF animal room was constant at 25°C and the air humidity was constant at 60%. The animal room is strictly disinfected once a day by a special person, and the animal cages and litter are replaced every 2 days. Nude mice drink water and eat freely in the animal room cage. The drinking water is homemade distilled water, and the diet is a special term for immunodeficiency animals.

### Self-Microemulsion of Triptolide

The solubility of TP in different oil phases, the surfactant, and the co-surfactant was determined and compared on the basis of a previous study. The middle-chain triglyceride (MCT) with better solubility was selected as the oil phase, the polyethylene-polyoxyethylene-oil (EL) was selected as the surfactant, and PEG-400 was selected as the co-surfactant. The blank self-microemulsion was also prepared. The emulsion time and the appearance of the microemulsion were used as indices for comparison.

### Central Composite Design and Response Surface Methodology

The above-mentioned screening process identified two factors that had a large influence on the properties of TP in the micro-emulsion [the ratio (X1) and Km (X2) of the oil phase]. The quality of the selected oil phase was 10–30% and the Km range was 1–2; the prescription optimization was continued according to the pre-experimental results in the earlier stage. Using the micro-emulsion particle size (Y1) and the dosage (Y2) as evaluation indices, the optimal prescription was predicted by two-factor five-level star-point design (21) The level of each factor is shown in Supplementary Table 1.

### Data Model Fitting and Effect Surface Analysis

According to the above experimental design results, the multivariate linear regression and quadratic polynomial equation fitting of each factor level were carried out using the Design Expert 8.0.6 software; subsequently, the three-dimensional effect surface map was drawn.

### Optimal Prescription Verification

The drug-containing self-microemulsion was prepared according to the mass ratio of the above-mentioned excipients (MCT 2.53 g; El 4.96 g; PEG 4002.51 g). After mixing evenly, 30 mg TP raw material was added followed by oscillation in water bath at 37°C for 24 h. After placing overnight, TP-SMEDDS was obtained by holding the mixture at 37°C for 1 h. Subsequently, the particle size and drug content of the prepared TP-SMEDDS were determined.

### Quality Evaluation of TP-SMEDDS

Newly prepared TP-SMEDDS was placed in the beaker and photographed with a digital camera to assess the shape. The particle size and distribution of TP-SMEDDS concentrate was 1 g, and 0.1 mol/L hydrochloric acid was added to 50 mL at 37°C. The particles were stirred uniformly at 100 r/min, and the particle size was determined by laser particle sizer.

Morphological observation: 50 mL 0.1 mol/L hydrochloric acid at 37°C was added to 1 g TP-SMEDDS concentrated solution and stirred evenly at 100 r/min with application of appropriate amount of copper mesh. Finally, the excess liquid was sucked with filter paper and the mixture dried. A drop of uranium peroxide acetate solution was added and dried naturally. Subsequently, it was examined under TEM (JEM- 1200EX). To determine the content of TP-SMEDDS, three batches were used. 1 mL was applied to 50 mL mobile phase and ultrasound for 5 min. Then accurately moving the diluent 2 to 10 mL with the mobile phase. The content of TP was determined.

### *In vitro* Release Test

Two milliliter microemulsion was obtained in a dialysate bag with molecular weight, the PBS (pH 7.4) was used as the medium, 1% Tween-80 was added as the cosolvent, and the temperature was set at 37°C and oscillated at 100 r/min. At 0, 0.3, 0.5, 0.75, 1, 1.5, 2, 3, 4, 5, 6, 8, 10, 12, 14, 5, 6, 8, 10, 12, 14, 20, 24 h, respectively, 1 mL of dialysate was taken and rapidly replenished with equal volume of pH 7.4 PBS buffer containing 1% Tween-80 at same temperature. The content of TP in the dialysate was determined by high performance liquid chromatography (HPLC). The cumulative release curve was drawn and the cumulative release percentage (Qn) was calculated.

### Inhibitory Effect on Xenograft Tumor of Human Gastric Cancer in Nude Mice

A transplantation tumor model was used to establish the logarithmic growth phase of MGC80-3 cells (22, 23). After trypsin digestion, the cell density was adjusted to 5 × 10^7^ cells/mL, and 0.2 mL was injected into the right axilla of each nude mouse subcutaneously (containing 1 × 10^7^ cells). Tumor formation was examined every other day after injection. When the mean tumor volume reached (56.71 ± 10.17) mm^3^, about 2 weeks, the model animals were randomly divided into 7 groups (5 nude mice in each group). These were: the model control group; high- (TP 0.6 mg/kg), medium- (0.4 mg/kg), and low-dose (0.2 mg/kg) TP-SMEDDS groups; and high- (TP 0.6 mg/kg), medium- (0.4 mg/kg), and low-dose (0.2 mg/kg) TP groups. The model control group was administered the same volume of saline once every 2 days. The tumor mass volume was measured every 3 days. The nude mice were killed 21 days after administration, the tumor mass was stripped and weighed, calculated according to the following formula: tumor volume = (tumor long diameter × tumor width^2^)/2. Tumor growth curve was drawn at the end of the experiment.

Tumor inhibition rate (IR) = model group average tumor weight-treatment average tumor weight/model group average tumor weight × 100%.

Tumor growth delay time = days required for tumor volume to reach 100 mm^3^ (or 200 mm^3^) in the treatment group minus days required for tumor volume to reach the same volume in the control group.

### Immunohistochemistry

The gastric cancer tissue were fixed in 10% buffered formalin and then the paraffin section were made by standardized steps. For immunohistochemical staining, after deparaffinization, the slices were subjected to antigen recovery in 0.01 M sodium citrate buffer at 125°C for 30 s, followed by 10 s at 90°C, and then subjected to the endogenous peroxidase inactivation by covering tissue with 3% hydrogen peroxide for 5 min (24). Then 10% goat serum was applied for block. 1:50-diluted anti-Ki-67 antibody was used for overnight incubation. After incubation with secondary antibodies, DAB solution was added for 3 min and counter stained with hematoxylin for 2 min. The expressions of Ki67 and p53 protein were detected and quantified using the Image-Pro Plus 6.0 software. The intensity of brown color was used as the observation index, and the optical density of the immunohistochemical results was analyzed to compare the IOD values between the groups.

### Statistical Analysis

All experimental data are expressed as mean ± standard deviation. The *t*-test was used to compare mean values between two samples. One-way ANOVA was used for multi-group comparisons of mean values. SPSS 17.0 was used for statistical analysis; *P* < 0.05 were considered indicative of statistical significance. GraphPad Prism6 was used for data presentation.

## Results

### Optimization of Self-Microemulsion of TP

**Figure 2:**
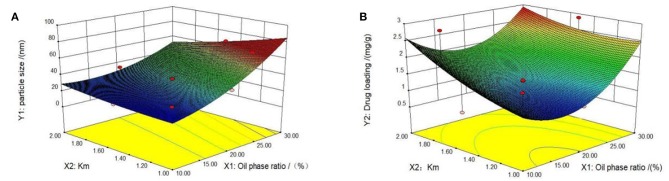
Three-dimensional response surface diagrams. Two influencing factors, oil phase mass ratio and km value, were identified by Design Expert 8.0.6 software. The effect surfaces of three-dimensional curves of particle size and drug loading of the response are shown **(A,B)**. The optimal formulation was determined based on the previous quadratic polynomial fitting equation and response surface diagram.

First, we tested the self-microemulsion of TP. The results showed that the self-microemulsion solution was in a better state and the self-emulsifying time was shorter when the mass ratio of oil phase was between 10 and 30% (Supplementary Table 2). Therefore, this proportion range of the oil phase was selected in the follow-up study for optimization. The particle size and drug content of microemulsion were determined by 13 experiments designed by central composite design and response surface methodology (Table 1). Next, we performed data model fitting and effect surface analysis. The three-dimensional effect surface is shown in Figure 2. The multivariate linear regression equation was: Y_1_ = 12.14010 + 220.39983 × X_1_ − 11.89697 × X_2_, *r* = 0.9278; Y_2_ = −0.61754 + 4.12547 × X_1_ + 0.92727 × X_2_, *r* = 0.5503. The multivariate linear regression equation revealed a low linear correlation coefficient, which indicates a poor linear correlation between the independent and dependent variable. Therefore, quadratic polynomial fitting was adopted (12).

**Table 1:** Central composite design and response.

**No**.	**X_**1**_ (oil phase mass percentage; %)**	**X_**2**_ (Km)**	**Y_**1**_ (grain size; nm)**	**Y_**2**_ (drug-containing amount; mg/g)**
1	12.93	1.15	27.05	1.58
2	12.93	1.85	24.28	2.81
3	27.07	1.15	67.45	1.53
4	27.07	1.85	44.54	2.28
5	10	1.5	22.31	0.82
6	30	1.5	67.57	2.88
7	20	1	39.12	1.00
8	20	2	33.54	1.45
9	20	1.5	33.71	1.33
10	20	1.5	33.43	1.27
11	20	1.5	35.22	1.31
12	20	1.5	35.22	1.28
13	20	1.5	35.43	1.24

The quadratic polynomial fitting equation was follows: Y_1_ = 5.60278 + 107.80933 × X_1_ + 6.80255 × X_2_ − 03.47545 × X_1_X_2_ + 1044.50919 × X_1_^2^ + 7.33186 × X_2_^2^, *r* = 0.9906.Y_2_ = 2.61159–21.41625 × X_1_-0.38649 × X_2_-84946 × X_1_X_2_ + 82.03979 × X_1_^2^ + 0.76122 × X_2_^2^, *r* = 0.9457. The correlation coefficients (*r*) of the quadratic polynomial fitting equation for particle size and drug content were 0.9906 and 0.9457, respectively, which indicated a good fit of the design model. The model can be used to predict and analyze the formula of tripterygiolol self-microemulsion. According to the prediction results, taking the smaller Y1 and the larger Y2 as the comprehensive objective, the optimal mass ratio of the excipients in the prescription of self-microemulsion was determined to be 31.168 nm of MCT:EL:PEG-400 = 25.3:49.6:25.1. The theoretical particle size and the optimal mass ratio of the excipients was 31.168 nm. The theoretical drug content was 2.981 mg/g.

To optimize and verify the prescription, we checked the validation results from the central composite design-effect surface optimization method (Table 2). We found that the deviation of particle size was 2.27 and the deviation of drug content was 2.38 (where the deviation = forecasted value minus actual value/predicted value ^*^ 100). The absolute deviation value for each index was small, which showed that the star design-effect surface optimization method had better prediction effect, and the binomial fitting degree was good. The obtained fitting equation can accurately describe the relationship between the factors and the index.

**Table 2:** Validation of central composite design-effect surface optimization.

	**Grain size (nm)**	**Drug content (mg/g)**
Predicted value	31.168	2.981
Actual value	30.46	2.91
Deviation (%)	2.27	2.38

### The Quality of TP SMEDDS

**Figure 3:**
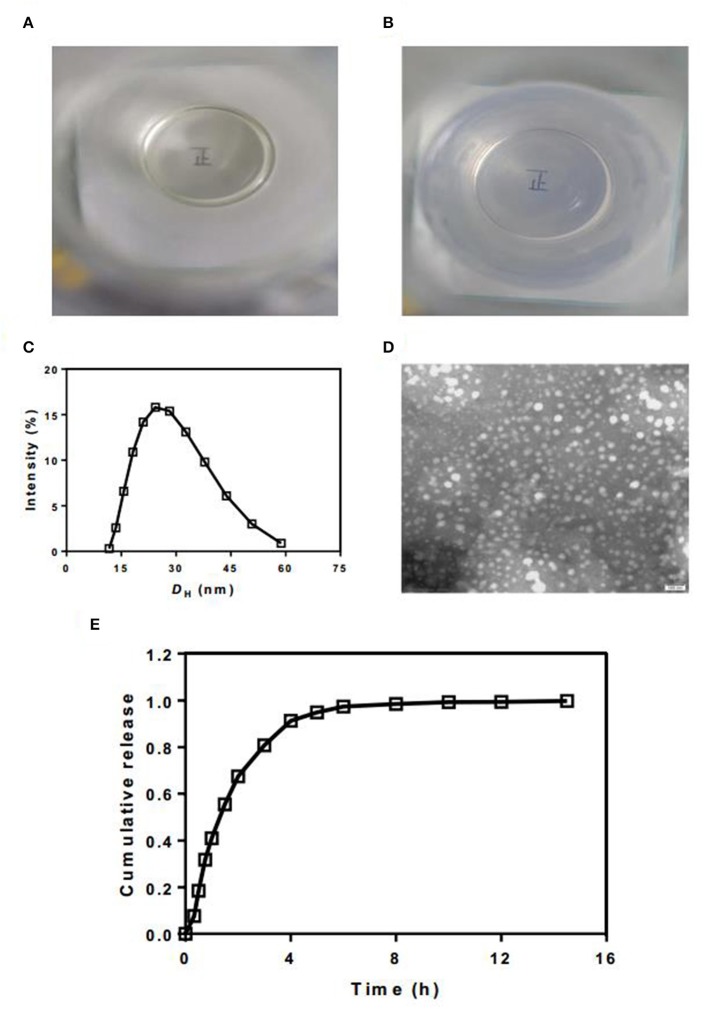
**(A,B)** The appearance of newly prepared TP-SMEDDS and hydrated TP-SMEDDS; **(C)** Particle size profile of TP-SMEDDS; **(D)** Transmission electron microscope photograph of TP-SMEDDS; **(E)** *In vitro* release profiles of TP-SMEDDS. TP-SMEDDS, triptolide-self-microemulsifying drug delivery system.

We then evaluated the quality of TP SMEDDS. The overlooking image of newly prepared TP-SMEDDS exhibited a clear and transparent liquid appearance (Figures 3A,B). After addition of 50 times water phase emulsion, the microemulsion transformed to high clarity and exhibited blue/white color. Through the colorless transparent beaker with microemulsion, we could clearly see the word “positive” written on the paper. To check the particle size and distribution, we used laser particle size measuring instrument. The results showed a mean particle size of 30.46 nm (Figure 3C). Moreover, the electron microscopic photographs showed that most of the microemulsion droplets formed after hydration were <50 nm in size, spherical in shape, and were uniformly distributed without adhesion (Figure 3D). Subsequently, we determined the drug content. The content of TP in the three batches of TP-SMEDD was 2.91 mg/kg, 2.94 mg/kg, and 2.84 mg/kg, respectively, which was in accordance with the requirements of TP. Finally, we carried out *in vitro* release test. The release time of triptolide from the microemulsion system in PBS solution at pH 7.4 was 6 h (Figure 3E).

### The Anti-tumor Effects of TP-SMEDD *in vivo*

**Figure 4:**
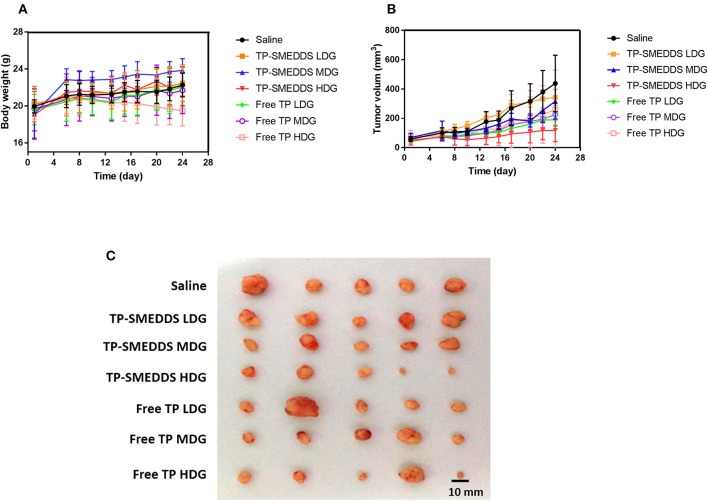
*In vivo* antitumor effect tested in MGC80-3-bearing mice (*n* = 5). TP-SMEDDS LDG and Free TP LDG were intragastric administration to mice at the dose of 0.2 mg/kg, TP-SMEDDS MDG and Free TP MDG were intragastric administration to mice at the dose of 0.4 mg/kg, TP-SMEDDS HDG, and Free TP HDG were intragastric administration to mice at the dose of 0.6 mg/kg, and 0.9% saline was used as control. **(A)** Trend of change in body weight of nude mice; **(B)** Growth curve of transplanted tumor in nude mice; **(C)** Representative morphology of transplanted tumors in nude mice of each group. TP-SMEDDS, triptolide-self-microemulsifying drug delivery system; LDG, low-dose group; MDG, medium-dose group; HDG, high-dose group.

In order to evaluate the *in vivo* anti-tumor effects of TP-SMEDD, we employed a xenograft model established with MGC80-3 cell transplantation. We first checked the general impact of different treatments on the xenograft mice. About 5 days after inoculation of MGC80-3 cells in nude mice, small nodules could be seen subcutaneously. The success rate of inoculation was >90%. At the 1st week after administration, the tumor-bearing mice in each group were well-proportioned with smooth fur and showed normal intake of water. There was no significant difference between the groups (Supplementary Table 3). On the 8th day, the diet of nude mice in each group was reduced with slowing down of weight gain. Compared with the control group, the side effects of high-dose TP group may be related to the loss of weight, decrease in food intake, slowness of action, and even death (Figure 4A). However, mice in TP-SMEDDS treatment groups exhibited normal eating behavior, flexible activity, smooth fur, and relatively small tumor volume.

Subsequently, we assessed the treatment effects. The growth curve shows no significant between-group difference with respect to tumor volume (*P* > 0.05). During the administration period, the tumor growth rate in the control group was faster and the volume of tumor in the control group was larger than that in the administration group (Figure 4B). On the 13th day, the average tumor volume in nude mice in the control group was 176.8 mm^3^ (Supplementary Table 4), while administration groups showed a significant inhibitory effect of TP on tumor growth. At the end of administration, the tumor volume in the high-dose TPSMEDDS group and low-dose group was significantly smaller than that in the control group (Figure 4B). There was also a significant difference between the high-dose TP-SMEDDS group (117.5 ± 76.54 mm^3^) and the high-dose TP group (258.82 ± 271.61 mm^3^), which indicated that the inhibitory effect of TP-SMEDDS on tumor growth was stronger than that of TP. The tumor volume in the high-dose TP-SMEDDS group (117.5 ± 76.54 mm^3^) was significantly smaller than that in the medium-dose administration group (315.67 ± 120.06 mm^3^). The results suggest that the self-microemulsion delivery system can improve the bioavailability of TP and enhance its anti-tumor effect in a concentration-dependent manner.

### Immunohistochemistry

After the mice were sacrificed, tumors were collected for further examination. Representative tumor morphology in each group is shown in Figure 4C. Tumor weight, tumor growth inhibition rate, and tumor growth delay time in each group are shown in Table 3. The results showed a dose-dependent inhibitory effect of TP on tumor growth. The tumor inhibition rate in the TP-SMEDDS group was significantly greater than that in the TP group (*P* < 0 05), and the anti-tumor effect exhibited a dose-response relationship.

**Table 3:** Tumor weight, tumor inhibition rate, and tumor growth delay time in nude mice of each group.

	**Saline**	**TP-SMEDDS LDG**	**TP-SMEDDS MDG**	**TP-SMEDDS HDG**	**Free TP LDG**	**Free TP MDG**	**Free TP HDG**
Transplanted tumor weight (g)	0.34 ± 0.25	0.31 ± 0.10	0.28 ± 0.11	0.18 ± 0.09^#^	0.25 ± 0.55	0.24 ± 0.18	0.20 ± 0.20^#^
Tumor control rate (%)	–	8.82	17.65	47.06	26.47	29.41	41.18
Tumor growth delay time (days)	–	0	0	14	9	7	4

*TP-SMEDDS, triptolide-self-microemulsifying drug delivery system; LDG, low-dose group; MDG, medium-dose group; HDG, high-dose group*.

# *P < 0.05*.

**Table 4:** Expressions of Ki67 and p53 in tumor tissues of each group.

	**Saline/10^5^**	**TP-SMEDDS LDG/10^5^**	**TP-SMEDDS MDG/10^5^**	**TP-SMEDDS HDG/10^5^**	**Free TP LDG/10^5^**	**Free TP MDG/10^5^**	**Free TP HDG/10^5^**
Ki67	2.26 ± 0.65	1.60 ± 0.86	0.99 ± 0.93^#^	1.34 ± 0.34^#^	2.19 ± 1.56	1.07 ± 0.74^#^	1.81 ± 1.57
p53	2.55 ± 1.76	1.29 ± 0.86	0.63 ± 0.40^#^	1.29 ± 0.93	2.78 ± 2.16	2.03 ± 0.94	2.04 ± 0.82

# * P < 0.05*.

**Figure 5:**
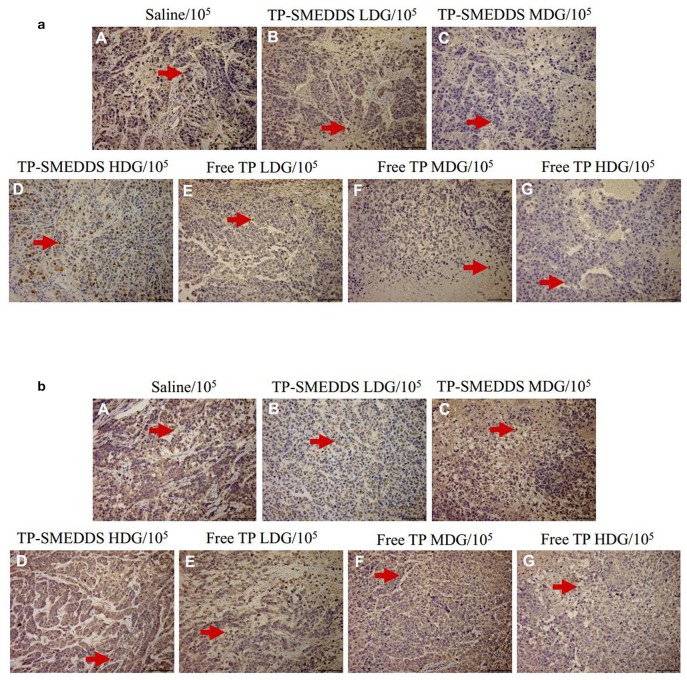
Tumor tissue sections of nude mice treated with different concentrations and dosage forms of TP-SMEDDS. And sections were well-prepared for observation of the pathological change separately under the microscope (×10^5^). TP-SMEDDS LDG and Free TP LDG were intragastric administration to mice at the dose of 0.2 mg/kg, TP-SMEDDS MDG and Free TP MDG were intragastric administration to mice at the dose of 0.4 mg/kg, TP-SMEDDS HDG and Free TP HDG were intragastric administration to mice at the dose of 0.6 mg/kg, and 0.9% saline was used as control. **(a)** Expression of Ki67 in tumor tissues; **(b)** Expression of P53 in tumor tissues. TP-SMEDDS, triptolide-self-microemulsifying drug delivery system; LDG, low-dose group; MDG, medium-dose group; HDG, high-dose group.

We then performed pathological evaluation of the tumors. We found that medium-dose TP-SMEDDS treatment significantly reduced the expressions of Ki67 and p53 as compared to that in the control and low-dose groups (Figures 5a,b). The results suggested that medium-dose TP-SMEDDS treatment has stronger inhibitory effect on tumor growth.

## Discussion

The prescription optimization and screening process typically involves assessment of the influence of multiple factors in order to optimize the results. In most cases, each index is influenced by a complex interaction between various factors, rather than a linear influence of a single factor (25, 26). In China, the research on prescription optimization and screening usually adopts the orthogonal design and the uniform design. The linear mathematical model is used for fitting. Therefore, the number of experiments is small, while the accuracy is not enough and the predictability is poor. In this study, the central composite design and response surface methodology was adopted to optimize the prescription (27). The method entails a step-by-step process. First, several factors that influence the prescription are identified as variables; then, the maximum and minimum values of each factor are determined according to the result of the single-factor experiment. Subsequently, according to the star point design factor level table, the number of experiments is determined. The relationship between the variable and the effect value is determined according to the results of the experiment. This method is more scientific and allows for better characterization of the interaction between various factors. This approach involves a small number of experiments and is characterized by high precision achieved via integration of mathematical and statistical methods. Previous reports have described the use of this method in the preparation process and for prescription screening optimization (21, 28, 29).

During the process of preparation of TP-SMEDDS, the composition of the initial formulation was obtained by selecting high solubility surfactants, cosurfactants, and oil-phase excipients. The prescription of TP-SMEDDS was optimized by the central composite design and response surface methodology. A polynomial model incorporating particle size and drug content, mass percentage of oil phase, and Km value was established. The effect surface of polynomial model was drawn. By optimization of effect surface curve using software, the optimal prescription composition of TP-SMEDDS was obtained. The results showed high reliability and predictability of the model. The evaluation indices of TP-SMEDDS according to this prescription were in accordance with the requirements. The current work lays a foundation for further research of this method for SMEDDS (21).

We evaluated the appearance, morphology, particle size and distribution, drug content, and release behavior of TP-SMEDDS. The average particle size of TP-SMEDDS (30.46 nm) was consistent with the size of the self-microemulsion preparation. In addition, the particle size of the prepared microemulsion was spherical, uniform in size and distribution, and exhibited no adhesion on transmission electron microscopy (TEM). The *in vitro* release experiment showed complete release of the prepared TP-SMEDDS in PBS solution at 6 h. The dissolution rate of TP was increased by the SMEDDS.

In this study, we also compared the inhibitory effects of water suspension and TP-SMEDDS on human gastric cancer cell line MGC80-3 xenografts. Compared with the model control group, TP-SMEDDS significantly inhibited the growth of transplanted tumor in nude mice. At the same dosage, the inhibitory effect on tumor volume growth in the TP-SMEDDS group was better than that in the TP groups. The tumor inhibition rate in the TP-SMEDDS group (47.06%) was higher than that in the TP group (41.18%). These findings suggest that the advantage of SMEDDS can make up for the deficiency of TP itself, promote its oral absorption, and enhance its antitumor activity.

The antigen Ki67 is a cell proliferation marker. Immunohistochemical staining for Ki67 can mark most of the cells outside the G0 phase; therefore, it is also referred to as the cell proliferation index (30–32). Wild type p53 protein is extremely unstable with a half-life of a few minutes. It has broad-spectrum tumor inhibition function. The stability and half-life of mutant p53 protein was increased resulting in its accumulation in the malignant cells (33, 34). The level of mutant p53 protein in tumor cells and abnormal transformed cells was shown to be 100 times higher than that in normal cells. Overexpression of p53 has been shown to be associated with metastasis, recurrence, and poor prognosis. P53 gene mutations have been implicated in several human cancers, such as liver cancer, breast cancer, bladder cancer, gastric, colon, prostate, soft tissue sarcoma, ovarian cancer, lymphocytic tumor, esophageal cancer, lung cancer, and osteosarcoma. In this study, two immunohistochemical markers, Ki67 and p53 (34–36), were used to demonstrate the possible anti-tumor mechanism of TP after self-emulsification. The results showed that TP decreased the expressions of Ki67 and p53 in tumor tissues before and after self-emulsification, which indicated that the anti-tumor effect of TP may be related to the inhibition of proliferation and metastasis of tumor cells (37–39). At the same time, the expression of TP at the same dose after self-emulsification was lower, which might be due to increase in its oral bioavailability and the enhancement of the inhibitory effect of SMEDDS on tumor cells.

## Data Availability Statement

The raw data supporting the conclusions of this manuscript will be made available by the authors, without undue reservation, to any qualified researcher.

## Ethics Statement

This study was carried out in accordance with the recommendations of the Animal Laboratory Committee of Zhejiang University. The protocol was approved by the Animal Laboratory Committee of Zhejiang University.

## Author Contributions

MX: experimental design, literature inquiry, Prescription design and optimization, Animal experiment, Data processing; JW: literature inquiry, Preparation of drug delivery system; LJ: Animal experiment; XJ: Cell experiment; JZ: Preparation of drug delivery system; MG: Experimental guidance; XC: Experimental guidance.

### Conflict of Interest

The authors declare that the research was conducted in the absence of any commercial or financial relationships that could be construed as a potential conflict of interest.
