# Supplementary material for: Development of Triptolide Self-Microemulsifying Drug Delivery System and Its Anti-tumor Effect on Gastric Cancer Xenografts
Source: Front Oncol. 2019 Oct 3;9:978. doi: 10.3389/fonc.2019.00978 (PMC6788343; doi:10.3389/fonc.2019.00978)
Supplement: Supplementary file 1 [file Table_1.docx]

Supplementary Table 1. Factors and levels for central composite design

| Factors | level | | | | |
| --- | --- | --- | --- | --- | --- |
|  | -1.141 | -1 | 0 | 1 | 1.141 |
| X_1_/% | 10 | 12.93 | 20 | 27.07 | 30 |
| X_2_ | 1 | 1.15 | 1.5 | 1.85 | 2 |
