# Supplementary material for: Development of Triptolide Self-Microemulsifying Drug Delivery System and Its Anti-tumor Effect on Gastric Cancer Xenografts
Source: Front Oncol. 2019 Oct 3;9:978. doi: 10.3389/fonc.2019.00978 (PMC6788343; doi:10.3389/fonc.2019.00978)
Supplement: Supplementary file 2 [file Table_2.docx]

Supplementary Table 2 Comparison of prescription SMEDDS with different proportions

| MCT  (%) | EL  (%) | PEG400  (%) | Self emulsifying time (s) | Appearance |
| --- | --- | --- | --- | --- |
| 10 | 45 | 45 | <10 | Clear blue emulsion, no precipitate after a week |
| 20 | 40 | 40 | <10 | Clear blue emulsion, no precipitate after a week |
| 30 | 35 | 35 | <10 | Blue-and-white milky, a little precipitate after a week |
| 40 | 30 | 30 | >180 | White and turbid without opalescence. A week after placement, a large amount of precipitate emerged. |
| 50 | 25 | 25 | >180 | White and turbid without opalescence. A week after placement, a large amount of precipitate emerged. |
| 60 | 20 | 20 | >180 | White and turbid without opalescence. A week after placement, a large amount of precipitate emerged. |

SMEDDS, self-microemulsifying drug delivery system
