# Supplementary material for: Development of Triptolide Self-Microemulsifying Drug Delivery System and Its Anti-tumor Effect on Gastric Cancer Xenografts
Source: Front Oncol. 2019 Oct 3;9:978. doi: 10.3389/fonc.2019.00978 (PMC6788343; doi:10.3389/fonc.2019.00978)
Supplement: Supplementary file 3 [file Table_3.docx]

Supplementary Table 3 Changes in body weight of nude mice in each group (n=5)

| Days | 1 | 6 | 8 | 10 | 13 | 15 | 17 | 20 | 22 | 24 |
| --- | --- | --- | --- | --- | --- | --- | --- | --- | --- | --- |
| Saline (g) | 19.94±0.71 | 21.06±1.25 | 21.26±1.18 | 21.18±0.93 | 21.26±0.91 | 21.48±1.03 | 21.56±1.19 | 21.50±1.23 | 21.84±1.28 | 22.26±1.28 |
| TP-SMEDDS LDG (g) | 20.2±1.92 | 20.78±1.86 | 20.86±1.74 | 21.40±1.05 | 21.40±1.72 | 21.50±2.06 | 21.74±2.08 | 22.12±1.90 | 22.12±2.13 | 22.46±1.98 |
| TP-SMEDDS MDG (g) | 19.12±2.72 | 22.84±1.17 | 22.74±1.05 | 22.80±0.93 | 22.86±0.95 | 23.12±1.20 | 23.44±1.34 | 23.34±0.82 | 23.68±1.11 | 23.84±1.27 |
| TP-SMEDDS HDG (g) | 19.04±2.52 | 21.47±1.32 | 21.58±1.24 | 21.57±1.18 | 21.38±1.11 | 22.23±0.85 | 21.77±1.51 | 22.67±1.51 | 21.97±1.81 | 22.12±2.10 |
| Free TP LDG (g) | 19.60±1.65 | 20.44±2.10 | 20.82±1.54 | 20.74±1.67 | 20.40±1.84 | 21.14±2.01 | 21.12±2.11 | 21.74±2.24 | 21.62±2.49 | 22.08±2.11 |
| Free TP MDG (g) | 19.70±2.41 | 20.66±2.80 | 21.04±2.68 | 21.02±2.07 | 20.80±2.41 | 21.08±2.21 | 21.00±2.07 | 21.70±2.72 | 21.34±2.71 | 21.68±2.53 |
| Free TP HDG (g) | 20.22±1.64 | 21.14±2.00 | 20.80±1.92 | 20.56±1.66 | 20.30±2.00 | 20.38±2.04 | 20.28±2.18 | 19.88±1.94 | 19.66±1.69 | 19.48±1.65^#^ |

# P < 0.05 versus control group

TP-SMEDDS, triptolide-self-microemulsifying drug delivery system; LDG, low-dose group; MDG, medium-dose group; HDG, high-dose group
