# Supplementary material for: Development of Triptolide Self-Microemulsifying Drug Delivery System and Its Anti-tumor Effect on Gastric Cancer Xenografts
Source: Front Oncol. 2019 Oct 3;9:978. doi: 10.3389/fonc.2019.00978 (PMC6788343; doi:10.3389/fonc.2019.00978)
Supplement: Supplementary file 4 [file Table_4.docx]

Supplementary Table 4 Changes of tumor volume in nude mice in each group

| Days | 1 | 6 | 8 | 10 | 13 | 15 | 17 | 20 | 22 | 24 |
| --- | --- | --- | --- | --- | --- | --- | --- | --- | --- | --- |
| Saline (mm^3^) | 59.23±16.26 | 100.7±34.85 | 108.6±29.65 | 111.2±21.47 | 176.8±68.95 | 188.4±61.59 | 269.0±118.4 | 316.8±118.9 | 379.7±146.6 | 438.1±192.4 |
| TP-SMEDDSLDG (mm^3^) | 53.77±10.07 | 110.00±19.81 | 128.10±31.57 | 148.77±29.85 | 205.99±11.95 | 223.28±7.054 | 289.62±32.23 | 310.93±33.98 | 333.02±62.64 | 343.24±53.64 |
| TP-SMEDDS MDG (mm^3^) | 68.83±28.78 | 116.29±64.79 | 97.13±32.68 | 110.55±28.46 | 132.36±36.43 | 160.17±36.79 | 195.20±39.24 | 181.88±41.16 | 254.22±83.58 | 315.67±120.06 |
| TP-SMEDDS HDG (mm^3^) | 47.42±29.36 | 75.16±31.41 | 60.18±42.87 | 54.52±41.53 | 64.28±41.73 | 75.46±43.05 | 93.68±63.51 | 105.4±73.72 | 116.9±62.89 | 117.5±76.54^#^ |
| Free TP LDG (mm^3^) | 42.85±8.124 | 76.23±20.99 | 80.14±14.16 | 92.49±16.39 | 99.71±6.223 | 105.8±16.16 | 131.3±12.45 | 160.9±14.72 | 188.0±11.58 | 190.4±43.83^#^ |
| Free TP MDG (mm^3^) | 51.97±10.13 | 70.76±23.56 | 75.70±18.19 | 81.22±19.01 | 99.47±33.80 | 118.6±40.47 | 157.2±43.63 | 178.2±55.01 | 195.1±56.57 | 226.2±74.66^#^ |
| Free TP HDG (mm^3^) | 72.89±44.33 | 81.43±29.78 | 80.45±20.84 | 92.21±43.03 | 129.01±108.79 | 124.23±109.29 | 180.45±183.15 | 196.08±192.52 | 226.02±196.00 | 258.82±271.61^#^ |

# P < 0.05 versus control group

TP-SMEDDS, triptolide-self-microemulsifying drug delivery system; LDG, low-dose group; MDG, medium-dose group; HDG, high-dose group
